# DOES PHYSICAL FUNCTION RESPONSE TO INTENTIONAL WEIGHT LOSS IN OLDER ADULTS VARY BY SEX-GENDER?

**DOI:** 10.1093/geroni/igz038.2532

**Published:** 2019-11-08

**Authors:** Kristen Beavers, Rebecca Neiberg, Daniel P Beavers, Eliza Dewey, Dalane Kitzman, Stephen Messier, Jack Rejeski, Stephen Kritchevsky

**Affiliations:** 1 Wake Forest University, Winston-Salem, North Carolina, United States; 2 Wake Forest School of Medicine, Winston-Salem, North Carolina, United States

## Abstract

The purpose of this study is to explore whether the effect of intentional weight loss on physical function in older adults varies by sex/gender. Individual level data from 1369 older, (67.7±5.4 years), obese (BMI: 33.9±4.4 kg/m2), adults (30% male, 21% African American) who participated in eight randomized controlled trials of weight loss were pooled. All studies were 5-6 months in duration and collected baseline demographic and pre/post gait speed (n=1296), short physical performance battery (SPPB; n=866), and grip strength (n=401) data. Treatment effects were generated by weight loss assignment [weight loss (WL; n=764) versus non-weight loss (NWL; n=605)], as well as categorical amount of weight change (high loss: >-7%, moderate loss: -7 to -3%, and weight gain/stability: <-3%). Analyses were adjusted for age, race/ethnicity, study, education, baseline BMI, and baseline value of the outcome measure of interest. Sex/gender stratified results were presented if the interaction term was p≤0.10. A sex/gender*weight loss assignment interaction was observed for SPPB (p=0.07), with women experiencing greater weight loss-associated improvement in SPPB score (WL: 0.42±0.08 versus NWL: 0.10±0.09; p=0.02) compared to men (WL: 0.30±0.11 versus NWL: 0.30±0.13). A sex/gender*weight loss amount interaction was observed for grip strength (p=0.05), with no difference observed across categories in women; however, greatest grip strength improvement was seen in men experiencing moderate weight loss compared to high loss and weight gain/stability categories. Weight loss-associated improvement in SPPB score is greater in women than men; grip strength gains in men are greatest among those achieving moderate weight loss.

